# Reference Gene Selection for Quantitative Real-time PCR Normalization in *Quercus suber*

**DOI:** 10.1371/journal.pone.0035113

**Published:** 2012-04-18

**Authors:** Liliana Marum, Andreia Miguel, Cândido P. Ricardo, Célia Miguel

**Affiliations:** Instituto de Biologia Experimental e Tecnológica (IBET) / Instituto de Tecnologia Química e Biológica-Universidade Nova de Lisboa (ITQB-UNL), Oeiras, Portugal; University of New England, Australia

## Abstract

The use of reverse transcription quantitative PCR technology to assess gene expression levels requires an accurate normalization of data in order to avoid misinterpretation of experimental results and erroneous analyses. Despite being the focus of several transcriptomics projects, oaks, and particularly cork oak (*Quercus suber*), have not been investigated regarding the identification of reference genes suitable for the normalization of real-time quantitative PCR data. In this study, ten candidate reference genes (*Act*, *CACs*, *EF-1α*, *GAPDH*, *His3*, *PsaH*, *Sand*, *PP2A*, *ß-Tub* and *Ubq*) were evaluated to determine the most stable internal reference for quantitative PCR normalization in cork oak. The transcript abundance of these genes was analysed in several tissues of cork oak, including leaves, reproduction cork, and periderm from branches at different developmental stages (1-, 2-, and 3-year old) or collected in different dates (active growth period *versus* dormancy). The three statistical methods (geNorm, NormFinder, and CV method) used in the evaluation of the most suitable combination of reference genes identified *Act* and *CACs* as the most stable candidates when all the samples were analysed together, while *ß-Tub* and *PsaH* showed the lowest expression stability. However, when different tissues, developmental stages, and collection dates were analysed separately, the reference genes exhibited some variation in their expression levels. In this study, and for the first time, we have identified and validated reference genes in cork oak that can be used for quantification of target gene expression in different tissues and experimental conditions and will be useful as a starting point for gene expression studies in other oaks.

## Introduction

The use of reverse transcription quantitative PCR (RT-qPCR) to assess transcript level has been widespread in plant biology. RT-qPCR is a sensitive, precise, easy and cost-effective method allowing the detection of low abundant mRNAs and slight variations in gene expression. It has also become the preferred method for the validation of microarray results.

To avoid bias, the use of reliable internal controls for RT-qPCR analysis is essential [1]. Genes required for the maintenance of basic cellular functions, such as *actin*, β-*tubulin*, *elongation factor*-1α and *18S rRNA* are commonly used as reference genes (RG) or internal controls. In theory, a RG is a gene with a constant level of expression in all cell types and under every experimental condition which may include developmental stages and biotic/abiotic stresses. However, a universal RG does not exist. In fact several studies reported that, according to the experimental conditions and species used, the level of expression of the commonly used RG can often be variable [2], [3], [4], [5], showing that these genes are differentially regulated among experimental conditions and plant species. Furthermore, it has been shown that the conventional use of a single gene for normalization may lead to relatively large errors in a significant proportion of samples [6], [7]. Currently, the use of multiple internal control genes is considered as an essential approach for an accurate normalization of data [5], [8], [9], [10]. Such an approach relies on the comparison of the mean variation of each gene relative to the mean variation of the other RG in order to obtain the best normalization factor. Statistical algorithms, such as geNorm [6] and NormFinder [11], were developed to facilitate the evaluation of potential RG expression stability under different experimental conditions. More recently, Hellemans et al. [12] also proposed the Coefficient of variation (CV) method as another powerful indicator of gene stability. Still, geNorm is the only tool that allows us to determine the minimum number of genes to be applied in normalization factor.

While the evaluation of expression stability of potential RG has been addressed under specific conditions for species such as *Arabidopsis* [2], [13], [14], barley, wheat and oat [15], rice [16], cotton [17], pea [7], flax [4], medick [18], tomato [19] and tobacco [20], in tree species only a few studies have been reported in poplar, spruce and longan [3], [10], [21], [22], [23]. Moreover, there is a general lack of information regarding the suitability of commonly used RG in the RT-qPCR analysis of target genes expressed in recalcitrant hardwood tissues such as wood, bark or cork.

Cork oak (*Quercus suber*), an evergreen tree characteristic of the Western Mediterranean (Portugal, Spain, Southern France, Italy, North Africa), has a remarkable capacity to produce suberose tissue, the phellem or cork [24], with unique properties that make it an excellent material for industrial applications. Due to the ecological and socio-economic significance of this species, large scale transcriptomic projects have been recently launched, targeting specific stress tolerance mechanisms and developmental processes such as cork differentiation [25], [26], [27], [28]. Therefore, the need for RT-qPCR approaches to determine, as accurately as possible, the transcript abundance of specific genes is evident. The expression level of target transcripts in cork, as measured by RT-qPCR, has already been studied by Soler et al. [29], but a thorough evaluation of the expression stability of RG was not reported.

In order to select the most suitable RG for gene expression quantification by RT-qPCR, we analysed several tissues of cork oak including leaves, reproduction cork and periderm from branches at different developmental stages or collected in alternate seasons. Ten potential RG involved in different biological roles, such as cytoskeleton structure [*Actin* (*Act*), *ß-Tubulin* (*ß-Tub*)], translational elongation [*Elongation factor-1alpha* (*EF-1α*)], carbohydrate metabolism [*glyceraldehyde-3-phophate dehydrogenase* (*GAPDH*)], chromosome organization, biogenesis and nucleosome assembly [*Histone 3* (*His3*)], chloroplast constitution [*Photosystem I psaH* (*PsaH*)], vesicle trafficking and endocytosis [*SAND family* (*Sand*)], protein modification process [*Ubiquitin* (*Ubq*)], protein kinase cascade [*Serine/threonine protein phosphatase* (*PP2A*) ] and intracellular protein transport [*Clathrin adaptor complexes medium subunit family* (*CACs*) ], were assessed using several statistical approaches for the normalization of data.

## Materials and Methods

### Plant material

Cork oak leaves, periderm tissues isolated from branches and reproduction cork were used for sampling at these locations. While reproduction cork was harvested from 3 trees growing in Coruche and São Brás de Alportel (Portugal), leaves and branches were collected from a single donor tree at Instituto Superior de Agronomia (Lisboa, Portugal). Young leaves and 1 to 3-year-old branches were collected during the active growth period in May 2010. Three-year-old branches were also collected during the active growth period in April and July 2010 and during the dormancy period in January 2010. Periderm tissues were isolated from branches by peeling off the external bark with a sterile scalpel. Reproduction cork was harvested during the debarking period in July 2009 and 2010. All the harvested tissues were immediately frozen by immersion in liquid nitrogen and stored at −80°C until further use.

### Total RNA extraction and purification

Frozen samples were ground to a fine powder in liquid nitrogen using a mortar and pestle. Total RNA was extracted following a protocol developed for grapevine [30] with minor modifications: (1) all centrifugations were performed at 13200 rpm; (2) after the addition of isopropanol the recovered nucleic acids were dissolved in 375 µl Tris-EDTA buffer (pH 7.5); (3) total RNA was precipitated with 140 µl of 8 M LiCl overnight at 4°C and (4) the final pellet was dissolved in 50 µl DEPC-treated water.

To remove any traces of genomic DNA contamination after RNA extraction, two different DNase treatments, DNase I (Qiagen) and TURBO DNase (Ambion), were tested according to the manufacturer's instructions. The RNA samples treated with DNase I were also purified using the RNeasy MinElute Cleanup (Qiagen). The integrity of the RNA samples was assessed by 1% (w/v) agarose gel electrophoresis with ethidium bromide staining. RNA concentration and the 260/280 and 260/230 nm absorbance ratios were determined using a ND-1000 Spectrophotometer (NanoDrop Technologies Inc., USA). The automated micro-capillary electrophoresis systems currently provide accurate resolution and sensitivity for analysis of RNA quality. By this fact, the first cork oak RNA samples extracted by the methodology described before were analysed by BioAnalyzer 2100 Agilent. However, as a routine procedure, the integrity of the majority of the RNA samples was assessed by 1% (w/v) agarose gel electrophoresis with ethidium bromide staining.

Absence of genomic DNA contamination was confirmed by performing PCR amplification using total RNA as template and primers designed for amplification of a 1069 bp DNA fragment (F:GGAGGCGTGGAAAGTGTTTA; R:ACTCAAACCCCAACGTAGCA) from the *glycerol-3-phosphate acyltransferase 5* gene (*GPAT5*) coding sequence (GenBank accession number:JN819185).

### First-strand cDNA synthesis and quality controls

cDNA was synthesized from 1.5 µg of total RNA using the Transcriptor High Fidelity cDNA Synthesis Kit (Roche) with the anchored-oligo(dT)_18_ primers according to the manufacturer's instructions. To standardize each biological replicate, the products from different cDNA synthesis reactions of the same RNA sample were combined.

In order to ensure reliable results in further steps, the RNA integrity was also checked by amplifying fragments in the 5′ and 3′ regions of *SHORT-ROOT* (*SHR*; GenBank accession number:JN819185) cDNA by qPCR. Primers were designed in the 5′ and 3′ regions of the *SHR* cDNA to amplify fragments of 177 and 193 bp, respectively (SHR_5′F: ATGGATACCTTGTTTAGGC; SHR_5′R: GGTTCAGTCCAATTTCGTTC; SHR_3′F: GTAGTGTCTAGAAGAAGACG; SHR_3′R: GCGTGTAAAGAAGGTACGC). The 3′/5′ ratio was determined according to the following equation: 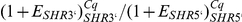 [31].

### Primer design

Ten candidate RG were evaluated in this study: *Act*, *CACs*, *EF-1α*, *GAPDH*, *His3*, *PsaH*, *Sand*, *PP2A*, *β-Tub* and *Ubq*. The RG were chosen based on their previous use as internal controls in gene expression studies of hardwood species such as *Q.suber*, *Populus* species and *V.vinifera*, and based on their consistent PCR amplification. RG sequences were obtained from the *Fagaceae* database, Fagaceae Genome Web (http://www.fagaceae.org/) and from GenBank. Primers were designed using Primer3 software [32] and PCR Primer Stats [33] taking into account the following criteria: annealing temperature of 60°C, GC content of 42–55% and primer length of 19–21 bp. The sequence accession numbers, the closest *Arabidopsis* homolog, as well as the primer sequences and amplicon size, are described in Table 1. To confirm the specificity of primer annealing the amplicons obtained after PCR amplification were sequenced, with the exception of *Act* and *β-Tub* already available in Genbank (EU697020 and EE743717, respectively). The amplicon sequences are presented in the supplementary data (Table S1).

**Table 1:** Description of the 10 candidate reference genes and primer sequences for RT-qPCR.

Gene abbreviation	Gene description	Accession number	*Arabidopsis* homolog locus	Primer sequences (forward/reverse)	Amplicon length (bp)
***Act***	Actin	EU697020	At5g09810	GCTGGTCGTGATCTAACTG/CTTTGCAGTCTCCAACTCCT	153
***CACs***	Clathrin adaptor complexes medium subunit family protein	ID6728500	At5g46630	TCTGGGAGAAGAGTGGCTACA/GAGCCACCATTCAAATCCT	175
***EF-1α***	Elongation factor 1-alpha	ID2007208	At5g60390	TTGTGCCGTCCTCATTATTGACT/TCACGGGTCTGACCATCCTT	75
***GAPDH***	Glyceraldehyde 3-phosphate dehydrogenase	ID6744996	At3g04120	ACCGACTTCATTGGTGACAG/AGATGCGATGTGGACAATCA	150
***His3***	Histone 3	ID6923956	At1g09200	GCTCTTCGAGGACACCAATC/TAAGCCCTCTCGCCTCTGAT	102
***PsaH***	Photosystem I psaH protein	ID6967182	At1g52230	CAGTTGCTCTGAAACCAAGG/CACAGCACCAGTCCTGAAGT	163
***PP2A***	Serine/threonine protein phosphatase 2A	ID6966573	At3g25800	GAGCCACTCTATCCGATTGC/GTCGTCATTGTTCTCGCTGA	162
***Sand***	Sand family protein	ID6961629	At2g28390	AGGATTGCAGGATTCGTATTG/GACCACCAATGCCAACAAA	204
***β-Tub***	β-Tubulin	EE743717	At2g29550	AAGAACATGATGTGCGCTGCT/TCCACCTCCTTGGTGCTCA	92
***Ubq***	Ubiquitin	ID3028522	At4g05320	CGAAGATCCAGGACAAGGAG/CAGGGCTTTTCACTCCTCAG	172

### qPCR conditions and PCR efficiency

The experiments were carried out in 96-well plates with a LightCycler 480 (Roche) using SYBR Green I Master (Roche) to monitor the PCR amplification. Reaction mixtures contained 10 µl of 2× SYBR Green I Master, 400 nM of each primer and 1.5 µl of cDNA as template, in a total volume of 20 µl. The following amplification program was used in all PCR reactions: 95°C for 10 min, 45 cycles of 10 s at 95°C, 10 s at 60°C and 10 s at 72°C. The specificity of each amplification reaction was verified by a dissociation curve (melting curve) analysis after the 45 cycles, by heating the amplicon from 65°C to 97°C. No-template controls were included for each primer pair.

For all RG studied 2 biological samples were used and the expression levels in each sample were based on 3 technical replicates. Leaf samples were used as calibrator to normalize the values between different plates.

Two different approaches were tested to determine the amplification efficiencies of the RG, using leaves as sample: a standard curve with a three dilution series calculated according to the equation (1+E) = 10^slope^ and the statistical algorithm Real-time PCR Miner. PCR efficiencies (E) for all the other samples were estimated with the Real-time PCR Miner algorithm [34] using the raw fluorescence data as input. Mean efficiency values were obtained for each biological replicate and were used to adjust quantification cycle (Cq) values for subsequent analysis.

### Experimental design and data analysis

Several RT-qPCR experiments were performed to analyse transcript levels in cork oak leaves, cork, periderm from 1, 2 and 3 year-old branches and periderm from 3 year-old dormant branches. For clarity samples corresponding to periderm from 1, 2 and 3 year-old branches together with cork will be referred to as developmental stage set and periderm from 3-year-old branches collected during active growth (May) *versus* dormancy (January) will be referred to as the seasonal growth sample set. Comparative analyses of all cork oak tissue samples, as well as individual analyses of the several sample types, were also performed.

RG transcript abundance in all the samples was determined by the Cq value or the number of cycles needed to reach a specific threshold level of detection in the exponential phase of the PCR reaction. Three statistical approaches were used to determine the stability of the candidate RG: geNorm v3.5 [6], NormFinder [11] and CV method [12]. The Cq values were converted into relative quantities to be used as input data for geNorm and NormFinder (only Cq<40 were used for analysis). The conversion was performed through the formula 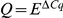, where E is the efficiency of the gene amplification for each primer pair (in each tissue) and ΔCq is the lowest Cq value as calibrator (which corresponds to the sample with the highest expression) minus the Cq value of the sample tested. The data obtained from each biological replicate were analyzed in separate. For the CV method the relative quantities were first transformed into normalized relative quantities (formula 15 as in Hellemans et al. [12]) and the CV was calculated using the standard error, through the formulas 17–19 Hellemans, et al. [12]). Finally, the normalization factor (NF) was based on the geometric mean of the best RG selected.

### Validation of RG analysis

One gene of interest putatively coding for a *glycerol-3-phosphate acyltransferase 5 (GPAT5)* (GenBank accession number: EE743865), was used to validate the selected RG. Primers were designed using Primer3 software [32] (GPAT5_F: GCTAGAGCGGTCTTGACAAAG; GPAT5_R: GACCTCATCAGCTCGCAAAT). The relative expression level of the target gene was determined in periderm tissues from 3-year-old branches collected in April and July 2010. The experimental procedure was the same as used in the selection of RG. For comparative purposes, the relative expression of the target gene was calculated with different normalization factors based on the geometric mean of the two most stable genes [lower M value, NF2(S)] and the two most unstable genes [higher M value, NF2(U)].

## Results

Data normalization using a set of reference genes (RG) is nowadays a current and crucial procedure when analysing the expression levels of target transcripts by RT-qPCR in different tissues or under different conditions. In the present study, the transcript abundance of 10 potential RG was assessed in cork oak by qPCR. A total of 36 cDNA samples including several tissue types and periderm tissues from branches under different developmental stages or collected during dormancy *versus* active growth period, were analysed.

### RNA quality

The assessment of RNA quality encompassed both its purity, characterized by the absence of protein and DNA contamination, and its integrity. All samples were analyzed spectrophotometrically and showed absorbance ratios at 260/280 and 260/230 nm above 1.8. Total RNA samples were also analysed in agarose gels showing well defined bands corresponding to the rRNA and absence of nucleic acid degradation. To confirm the absence of contaminating genomic DNA, positive and no RT controls were used in *GPAT* amplification. The DNase I (Qiagen) treatment proved inefficient for the complete removal of genomic DNA from total RNA extracted from cork oak tissues. However, the Turbo DNase (Ambion) proved effective in the removal of DNA contamination, since gene amplification was obtained only from reverse transcribed samples.

The RNA integrity was checked by performing a 3′∶5′ assay according to Nolan et al. [31]. This assay gives an indication of the mRNA integrity, since in most cases the RNA degradation starts in the 5′-end region. In general, while a 3′∶5′ ratio close to 1 means that the percentage of full-length transcripts in the sample is high, a ratio higher than 5 suggests degradation in the RNA samples. In our study the obtained 3′∶5′ ratios were close to 1 with primer efficiencies of 1.8 and 1.9. These results showed that total RNA samples used for RT-qPCR analyses were pure, non-degraded and free of DNA contamination.

### qPCR experiments and PCR efficiency

Specificity of amplification of the several transcripts was supported by the analysis of melting curves and by gel electrophoresis, showing a single PCR amplification product with the expected size for each gene (Fig. S1) and further confirmed by amplicon sequencing. The PCR efficiency (E) of each primer pair was first calculated in cork oak leaves through the standard curve method and then compared with the E value obtained through the statistical algorithm PCR Miner. According to Czechowski et al. [14] both methods give similar amplification efficiencies. In our study using cork oak leaves, the E values obtained by both methods were similar (Table S2). However, the standard curve method is time consuming, requiring the production of repeatable and reliable standards [35], with no errors from contamination or sample dilution. Moreover, this method relies on the assumption that the PCR efficiency of each amplicon is constant in all samples, which rarely can be achieved in real experiments [34], strongly influencing the Cq analysis [36], [37]. Therefore, Real-time PCR Miner algorithm [34], using the single raw fluorescence data as an input, was the chosen method to calculate the PCR efficiency for each primer pair in each tissue type (Table 2).

**Table 2:** PCR amplification efficiency of each primer pair.

	Tissue/stage					
Genes	Leaves	1^st^B	2^nd^B	3^rd^B	3^rd^DB	Cork
***Act***	1.93±0.03	1.96±0.02	1.94±0.02	1.90±0.02	1.91±0.02	1.94±0.03
***CACs***	1.93±0.02	1.97±0.02	1.94±0.01	1.94±0.02	1.97±0.02	1.95±0.02
***EF-1α***	1.92±0.02	1.95±0.02	1.92±0.02	1.96±0.02	1.96±0.02	1.93±0.02
***GAPDH***	1.98±0.02	1.96±0.01	1.91±0.02	1.92±0.03	1.90±0.01	1.94±0.02
***His3***	1.91±0.03	1.95±0.01	1.94±0.02	1.94±0.02	1.95±0.03	1.92±0.03
***PP2A***	1.95±0.01	1.92±0.02	1.97±0.02	1.96±0.02	1.91±0.04	1.97±0.02
***PsaH***	1.93±0.04	1.89±0.02	1.89±0.03	1.92±0.03	1.94±0.03	1.84±0.00
***Ubq***	1.94±0.02	1.91±0.02	1.92±0.01	1.93±0.02	1.94±0.03	1.86±0.01
***Sand***	1.92±0.02	1.92±0.02	1.95±0.03	1.91±0.01	1.91±0.03	1.93±0.05
***ß-Tub***	1.99±0.03	1.99±0.02	1.97±0.02	1.95±0.01	1.95±0.03	1.96±0.04

Efficiency values obtained after the amplification of each candidate RG (*Act, ß-Tub, , EF-1α, GAPDH, His3, PsaH, Sand, Ubq, PP2A, CACs*) in leaves, periderm from 1, 2 and 3-year-old branches (1^st^B, 2^nd^B and 3^rd^B), periderm from 3-year-old branches in the dormancy period (3^rd^DB) and cork, estimated with the Real-Time PCR Miner algorithm.

The 10 potential RG tested (*Act, ß-Tub, EF-1α, GAPDH, His3, PsaH, Sand, Ubq, PP2A and CACs*) were successfully amplified in cork oak tissues. The efficiency values (E) were calculated as the mean values obtained from the technical and biological replicates (Table 2), and used to adjust Cq values for subsequent analysis. Primer efficiencies were higher than 1.9 for all the experiments, except for *Ubq* in cork and for *PsaH* in periderm from 1 and 2-year-old branches and cork, where the values varied between 1.84 and 1.89 (Table 2). Altogether, these results confirm that the selected primers accurately amplify the potential RG.

**Figure 1:**
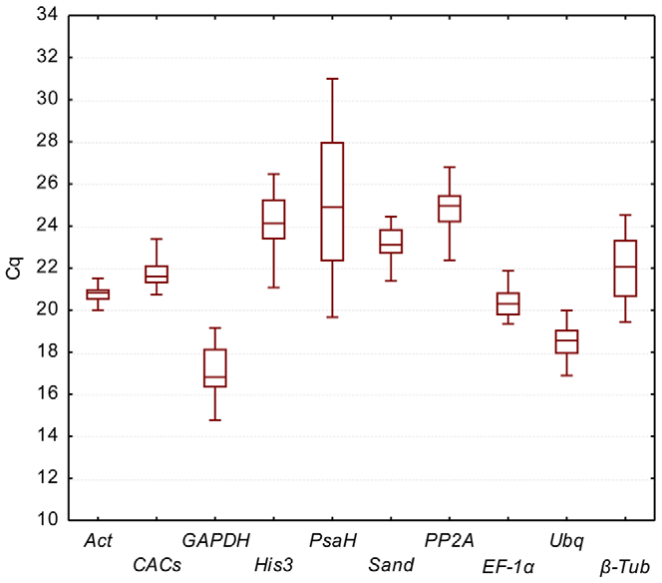
Range of Cq values of the candidate reference genes obtained for all cDNA samples. Each box corresponding to *Act*, *CACs*, *EF-1α*, *GAPDH*, *His3*, *Ubq*, *PsaH*, *Sand*, *PP2A* and ß-*Tub* indicates the 25% and 75% percentiles. Whiskers represent the maximum and minimum values. The median is depicted by the line across the box.

The calculation of mean Cq values for the ten RG in all cDNA samples (Fig. 1) showed a range of variation from 14.8 to 31.0. *GAPDH* displayed the most abundant transcript level, while *PsaH* was the less abundant. Based on the interquartile range (25–75% percentiles) for Cq values, the lower Cq dispersion was observed for *Act*, *CACs*, *EF-1α*, followed by *Ubq*, *Sand* and *PP2A*.

### Stability analysis

In order to identify and rank the most suitable RG based on their expression stability, three different statistical approaches, geNorm, NormFinder and CV method, were tested. For all analyses, the Cq values were transformed into relative quantities using the ΔCq method, and the amplification efficiencies of the RG were calculated by PCR Miner algorithm.

**Figure 2:**
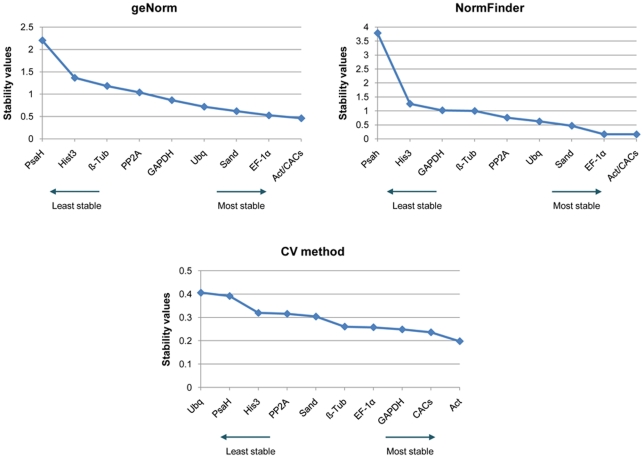
Stability values of candidate reference genes (RG) calculated by different statistical methods using all cDNA samples. Ranking of each RG (*Act*, *CACs*, *EF-1α*, *GAPDH*, *His3*, *Ubq*, *PP2A*, *PsaH*, *Sand*, *β-Tub*), calculated by geNorm, NormFinder and CV method, for all tested samples [leaves, branches (1^st^, 2^nd^, 3^rd^ active, 3^rd^ dormancy) and cork].

When using geNorm algorithm the candidate RG were ranked according to their expression stability measure (M), which represents the average pairwise variation of a particular gene with all other control genes. The stability values are reached after stepwise exclusion of the worst-scoring RG. Considering the data obtained from all samples, *Act* and *CACs* were the most stable genes (lowest M value of 0.462), followed by *EF-1α* (M value of 0.525) (Fig. 2). Hellemans et al. [12] recommended a stability measure threshold lower than 1 to ensure the most stable genes are selected. In our study six of the genes showed an M value lower than 1. The highest M value (2.203) was observed for *PsaH*, the most unstable gene, which can be explained by the lower expression levels observed for this gene in some of the tissues, namely cork and a few periderm samples.

A similar ranking of the tested genes was obtained when expression stability was analysed through the NormFinder algorithm taking into account all cDNA samples (Fig. 2). The three most stable (*Act*, *CACs* and *EF-1α*) and the two least stable (*PsaH* and *His3*) candidate genes were the same as identified by geNorm. When using the CV method, *Act* and *CACs* were also the best performing genes, but a different ranking of the remaining candidate RG was obtained (Fig. 2).

**Figure 3:**
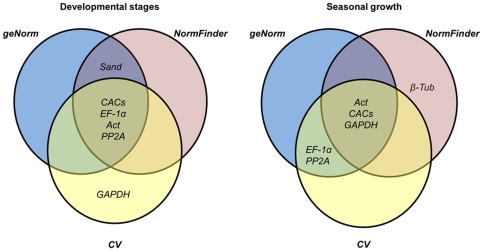
Venn diagram showing the most stable genes identified by the geNorm, NormFinder and CV method. The most stable genes were identified using data from the developmental stage and seasonal growth sample sets.

The analysis of the developmental stage data set by geNorm and NormFinder also revealed a similar ranking of the tested RG, while in the seasonal growth data set analysed by NormFinder and CV method, the most and less stable genes were similar but the intermediate rank positions differed (Fig. S2). Combining the three statistical approaches in the analysis of the developmental stage and seasonal growth data sets, the *Act*/*CACs*/*EF-1α*/*PP2A* and *Act*/*CACs*/*GAPDH* clusters represented the most stable genes in each set, respectively (Fig. 3).

However, when performing separate analysis of specific tissues, developmental stages or active growth *versus* dormancy, less uniform results were obtained (Table 3). For instance, in the active growth *versus* dormancy, *CACs*/*GAPDH*, and *EF-1α*/*Sand* can be used as good RG according to geNorm, while NormFinder selected *CAC*s/*GAPDH* and *Ubq* and CV method selected *CACs* and *Act* as the most adequate for data normalization.

**Table 3:** Stability values for the candidate RG in individual sample types.

Method	Tissue/stage	*Act*	*CACs*	*EF-1α*	*GAPDH*	*His3*	*Ubq*	*PsaH*	*Sand*	*PP2A*	*ß-Tub*
**geNorm**	Leaves	0.023	0.157	**0.008**	**0.008**	0.485	0.065	0.130	0.252	0.302	0.013
	1^st^B	0.030	0.087	0.009	0.194	0.138	0.068	0.002	**3.38E-04**	0.272	**3.38E-04**
	2^nd^B	0.098	0.276	**0.022**	0.255	0.145	0.059	0.843	0.209	0.432	**0.022**
	3^rd^B	0.167	**0.050**	0.218	**0.050**	0.288	0.415	1.086	0.590	0.080	0.686
	3^rd^DB	0.458	0.396	**0.047**	0.194	1.126	0.631	1.967	**0.047**	1.508	0.528
	Cork	0.548	0.608	0.679	0.472	0.233	0.308	0.214	0.086	**0.052**	**0.052**
**NormFinder**	Leaves	0.065	0.317	0.105	0.096	0.841	**0.037**	0.311	0.197	0.207	0.118
	1^st^B	0.093	0.006	0.041	0.309	0.176	0.006	0.014	**1.17E-04**	0.402	**1.17E-04**
	2^nd^B	0.034	**0.009**	0.144	**0.009**	0.039	0.247	1.722	0.031	0.840	0.173
	3^rd^B	0.036	**0.017**	0.036	**0.017**	0.239	0.433	1.858	0.840	0.024	0.878
	3^rd^DB	0.467	0.571	1.190	0.946	1.153	**0.116**	2.616	1.152	1.609	0.163
	Cork	0.353	0.467	0.658	0.338	0.521	**0.090**	0.517	0.292	0.174	0.224
**CV method**	Leaves	0.145	**0.038**	0.131	0.186	0.382	0.422	0.352	0.280	0.323	0.264
	1^st^B	0.182	0.253	0.193	**0.115**	0.289	0.273	0.249	0.219	0.319	0.201
	2^nd^B	0.141	0.182	0.159	0.163	**0.129**	0.216	0.440	0.170	0.320	0.216
	3^rd^B	0.167	**0.055**	0.211	0.257	0.127	0.377	0.444	0.365	0.207	0.352
	3^rd^DB	**0.107**	0.182	0.356	0.285	0.442	0.167	0.470	0.325	0.356	0.157
	Cork	0.345	0.377	0.395	0.299	0.298	0.325	0.485	0.348	0.227	**0.207**

The stability values for *Act*, *β-TUB*, *EF-1α*, *GAPDH*, *His3*, *PsaH*, *Sand*, *Ubq*, *PP2A*, *CACs* in leaves, periderm from 1, 2 and 3 years-old branches and cork (1^st^B, 2^nd^B, 3^rd^B) or from 3-year-old branches collected in alternate seasons corresponding to active growth *versus* dormancy (3^rd^B, 3^rd^DB) and cork, were calculated by geNorm, NormFinder and CV method. The values in bold refer to the most stable genes.

On the other hand, according to Hellemans et al. [12], the mean stability values (M) and the mean CV for heterogeneous sample panels should be within the M≤1 and CV≤0.5 ranges. Taking into account all cDNA samples, our results completely match these criteria. The obtained M values ranged from 0.462 to 0.866, except for the four less stable genes (*PP2A*, *β-Tub*, *His3* and *PsaH*), while CV values were in the range of 0.198–0.406. For homogeneous sample panels Hellemans et al. [12], consider a different range, M≤0.5 and CV≤0.25, for selecting the best RG. The M and CV values for the most stable RG selected from the separate analysis of the developmental stages set, seasonal growth set and the individual analysis of each sample type, also fits the proposed value ranges (Table 3; Fig. S2).

**Figure 4:**
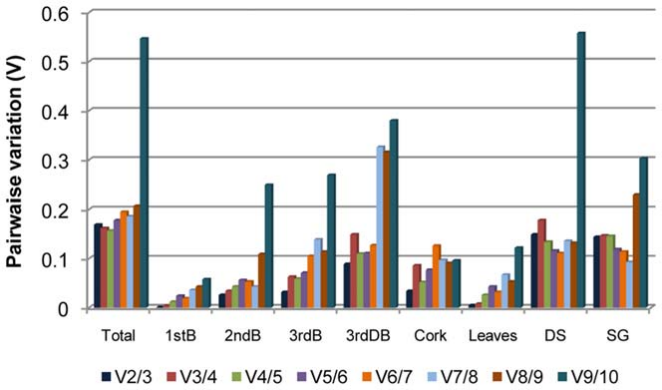
Determination of the optimal number of reference genes for normalization according to geNorm software. Pairwaise variation (V_n/n+1_) analysis between the normalization factors NF_n_ and NF_n+1_, carried out for all the samples (Total), individual samples [leaves, periderm from 1-year-old (1stB), 2-year-old (2ndB) and 3-year-old branches during active growth (3rdB) or dormancy (3rdDB) and cork], developmental stage sample set (DS) and seasonal growth sample set (SG).

The optimal number of RG used for data normalization was determined through the pairwise variation (V_n/n+1_), using the geNorm algorithm [6]. This is calculated between the two sequential normalization factors, NF_n_ and NF_n+1_ for all the samples under analysis and reveals the effect of adding an (n+1)^th^ gene, indicating whether the inclusion of an extra reference gene adds stability to the normalization factor. A small variation means no significant effect on the normalization by the addition of another gene. Our study reveals that all V_n/n+1_ values were bellow 0.15, except when the analysis was performed using the data from all the samples (Fig. 4). Although Vandesompele et al. [6] recommended a cut-off value of 0.15 (bellow which the addition of new gene is not required), this should not be considered as a strict threshold [38] and several subsequent studies have reported higher cut-off values of V_n/n+1_ [8], [39], [40]. Our data shows a small variation between V_2/3_(0.169) and V_3/4_(0.162), when all the samples were analysed together, suggesting that the addition of a third gene has no significant effect on the normalization factor.

### RG validation

The conclusions from the analyses described above were applied to quantify the transcript level of a gene of interest, *glycerol-3-phosphate acyltransferase 5 (GPAT5)*. The quantification of transcripts was performed in periderm tissues from 3-year-old branches collected in the spring (April) and summer (July), to validate the RG selected by the different statistical methods. The seasonal variation in *GPATs* transcript level had been previously evaluated in cork oak by Soler et al. [29] using RT-qPCR.

**Figure 5:**
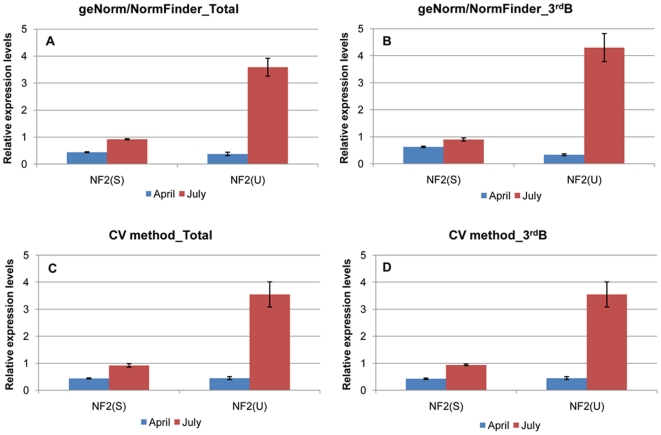
Validation of the reference genes (RG). Relative expression levels of *GPAT5* in periderm from 3-year-old branches collected in spring (April) and summer (July). Normalization factors were calculated with RG obtained in the analysis of data from all samples (Total) by geNorm/NormFinder (A) and CV method (C) or data from periderm of 3-year-old branches (3^rd^B), also in geNorm/NormFinder (B) and CV method (D). The normalization factors were based on the geometric means of the two most stable genes [NF2(S)] and the two most unstable [NF2(U)].

Different RG combinations were tested in order to assess whether the use of the different normalization factors (NF) obtained by geNorm, NormFinder and CV method had an impact on the transcript quantification results. The several NF were calculated taking into account the stability of the RG as inferred by different statistical methods (geNorm, NormFinder and CV method) and the type of data used for the analysis. Thus, the NF were determined using the two most stable, NF2(S), and the two most unstable genes, NF2(U), identified by geNorm and NormFinder, when analysing (1) data from all the samples [NF2(S) (*Act*; *CACs*) and NF2(U) (*His3*; *PsaH*)] and (2) data from periderm tissues of 3-year-old branches [NF2(S) (*CACs*; *GAPDH*) and NF2(U) (β-*Tub*; *PsaH*)] (Fig. 5). The NF were also calculated using the two most stable as well as the two most unstable genes identified by the CV method, when analysing data from all the samples [NF2(S) (*Act*; *CACs*) and NF2(U) (*PsaH*;*Ubq*)] or data from periderm of 3-year-old branches [NF2(S) (*CACs*; *GAPDH*) and NF2(U)(*Ubq*; *PsaH*)] (Fig. 5).

When the *GPAT* expression level was calculated with the NF2(S) obtained by the several statistical methods, a small variation (<0.5-fold) between the two seasons (spring and summer) was observed. However, important changes in the relative expression levels (≥3 fold) were obtained when the several NF2(U) were used.

**Figure 6:**
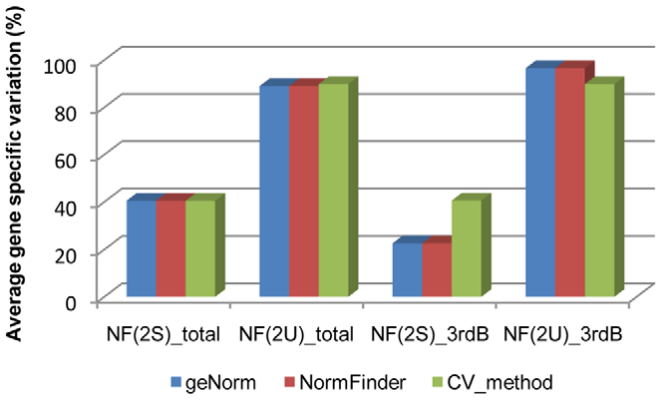
Average *GPAT5* gene specific variation. Determination of the coefficient of variation in percentage, for the two most stable, NF(2S), and the two most unstable genes, NF(2U). This analysis was performed for the RG selected from all tested samples (total) and from periderm from 3-year-old branches (3rdB), using three statistical methods (geNorm, NormFinder and CV method).

The different NF used also enabled us to calculate the average gene specific variation based on the most stable and unstable genes identified from the two type of data used for the analyses. The smallest average gene-specific variation (22.66%) was obtained with NF2(S) (*Act*; *CACs*), calculated by geNorm and NormFinder, for the data from periderm samples (Fig. 6). The highest gene-specific variation, 89% and 96%, was obtained with the NF calculated with the most unstable genes from geNorm, NormFinder and CV method, using data from all the samples and from periderm samples, respectively (Fig. 6).

## Discussion

Gene expression can vary across tissues or cell types as well as developmental and physiological stages. However, the genes commonly referred to as housekeeping function genes (HK) required to maintain basic cellular functions, are expressed in all metabolically active cells or tissues, being critical to the activities that must be carried out for successful completion of cell cycle [41]. Molecular characterization studies have even pointed to characteristic features of HK genes such as a lower degree of conservation of their promoters when compared to those of non-HK genes, and a higher density of SSRs in their 5′-UTRs [42], [43]. Due to their role, HK genes have been widely chosen as valuable controls (like RG) in gene expression analyses. Although HK genes continue to be often used, several studies showed that the HK genes are not necessarily expressed at the same level in all tissues [4], [44], [45]. Therefore, it is recognized that a careful choice of RG supported by experimental evidence is essential to obtain a reliable normalization of gene expression data for accurate quantification of transcript levels.

Although some authors still use RG from model plants, several studies have demonstrated that such genes are frequently unstable. For example, the *GAPDH* gene, involved in basic cellular functions and often assumed to have a uniform expression pattern, is one of the most stable genes in barley, oat and grapevine [15], [30] but in *N. tabacum* it proved to be much less stable [20]. Furthermore, it has been advocated that even for the same type of samples the RG can vary between experiments and laboratories, which can lead to misleading results. Nevertheless, the experimentally induced variations (samples, operator and instruments) can be strongly reduced by implementing a robust methodology.

In this study, the evaluation of ten candidate RG in cork oak samples encompassing different tissues, developmental and seasonal growth stages, was performed following a careful experimental design using several controls for checking RNA quality and integrity and PCR efficiency and specificity. Furthermore, the consistency of the best-scoring RG was tested by several statistical approaches available for this purpose. One of the critical steps addressed in the experimental procedures was to assure the high quality of total RNA isolated from cork oak recalcitrant tissues such as periderm and cork, containing highly suberized cells and high contents of secondary metabolites. This issue was successfully overcome by the use of an optimized RNA extraction protocol and a specific DNAse treatment. The RNA isolation method described here had been previously optimized for cork oak tissues and its effectiveness checked by several available methods including analysis in the Agilent 2100 Bioanalyzer (unpublished results). Although, automated micro-capillary electrophoresis systems currently provide higher resolution and sensitivity for analysis of RNA quality than agarose electrophoresis, the combination of several procedures namely spectrophotometric analysis in the NanoDrop, gel electrophoresis and the 3′∶5′-assay used in this work, was adequate for checking the quality of RNA samples isolated through this well established isolation procedure.

As expected, the variation in expression stability of the ten RG tested in our study suggests there is no single RG that can be used for a diversity of cork oak samples. The use of multiple RG that are stable under a given experimental condition was the chosen method for the normalization of RT-qPCR, in agreement with previous reports in species such as *Arabidopsis*, wheat, barley, oat, tomato, tobacco, pea, cucumber and poplar [5], [7], [9], [10], [19], [20], where a similar strategy has proved efficient for the relative quantification of a target gene.

Many of the RG used until now have been selected based on data compiled from microarray databases. However, Czechowski et al. [14] showed that, depending on the specificity of the database used to assess the expression stability, the ranking of the RG genes can change. In order to find adequate RG, several analysis tools have been developed [6], [11], [12], [46] but the question of which procedure is the most suitable remains open. From the different statistical approaches used in this work to analyse gene expression stability, the geNorm and NormFinder algorithms generated a similar RG ranking when considering data from all the samples (heterogeneous sample panel), while the ranking obtained with the CV method was slightly different. Although *CACs* and *Act* were within the best-scoring RG by any of the methods, *EF-1α* was ranked as one of the most stable genes by geNorm and NormFinder but not by the CV method. *Act* and *EF-1α* have been traditionally used as RG namely in *N. tabacum*, *V. vinifera* and *E. ulmoides*. The high stability of *CACs* has also been described in *Arabidopsis* time-course experiments(Hong et al., 2010), in *Cucumis sativus* subjected to abiotic stress and exogenously applied growth regulators [5], in different plant structures of *Fagopyrum esculentum* [9], and in vegetative and reproductive organs of *Vaccinium sp* [47]. In fact, this gene is involved in a number of essential cellular processes, including membrane trafficking, protein sorting and endocytosis, and it has been identified in *Arabidopsis* as being among the five most stable genes. On the other hand, *EF-1α*, one of the genes with a lower M value in our study, was shown to be quite unstable in *Arabidopsis* [14] and in *Salvia miltiorrhiza* [48] when geNorm algorithm was used, and also in barley, oat and wheat [9] when three different algorithms were applied. These results confirm that a universal reference gene does not exist, highlighting the need to evaluate commonly used RG for a particular species or condition. When the expression stability was analysed separately for each sample set, the ranking of the RG stability was not uniform. Some of the variation in expression levels may be due to the role of the RG in specific tissues. For example, the role of *PsaH* in the chloroplast as a component of photosystem I could explain the low expression levels found for this gene in some of the tested tissues such as cork. On the contrary, *β-Tub* was one of the most stable genes in cork. In fact, *β-Tub* had already been used as a RG in the quantification of cork transcripts by Soler et al. [29].

Despite the differences we found in the RG ranking when using different statistical approaches, there is a general agreement among the methods for the selection of the most stable genes. The geNorm algorithm is based on the geometric averaging of multiple genes and it has been the most used method [2], [3], [4], [7], [17], [20], [48] due to its simplicity and robustness in the calculation of the NF. The CV method is another powerful indicator which represents the variation of the normalized quantities across the tested samples while the NormFinder allows estimation, not only of the overall variation of the candidate gene, but also of the variation among sample subgroups. According to Andersen et al. [11], NormFinder is more effective in avoiding the effect of gene co-regulation because it takes into account the intra and inter-group variation. The geNorm software has the advantage of indicating the minimal number of RG required for data normalization through the pairwise variation tool. The use of an increased number of RG in the normalization can improve the reliability of a study, but it is time-consuming and more expensive and thus, a trade-off between the gain in accuracy and the costs and time involved needs to be carefully balanced. In the analysis of our data, the determination of the pairwaise variation of two sequential normalization factors (V_n/n+1_) using the geNorm software, indicated that 2 was the minimum number of RG to be included in the normalization for all the analysed sample sets. The inclusion of a third gene does not add any significant contribute to the calculation of the normalization factor.

Based on previous studies for determination of seasonal variance in transcript abundance of *GPAT5* in cork tissues, we chose this gene as a target to validate the RG selected in this study. *GPAT5* is involved in suberin biosynthesis, one of the main compounds of cork [49] and it has been identified in a EST collection showing a high and specific expression in the suberin-rich phellem of the cork oak tree [50]. When *GPAT5* expression was measured in periderm tissues from 3 year-old cork oak branches no significant variation in transcript abundance was found between April and July, which was in accordance with previous results reported by Soler et al. [29]. The importance of selecting the most stable genes to calculate the NF was evidenced by the huge difference observed in the relative expression levels when NF was calculated with the most stable genes *versus* the most unstable ones. The genes selected by geNorm and NormFinder, when data from the periderm of 3-year-old branches were used, seem to be more reliable to quantify the relative expression levels when comparing to the CV method, as judged by the lowest average gene-specific variation obtained with NF2(S) (*Act*; *CACs*).

This study is the first attempt to identify RG in several cork oak tissues. We concluded that *Act* and *CACs* were the most stable genes even when considering heterogeneous sample sets, and these were further validated in the transcript quantification of a target gene. These results should be a solid starting point to analyse the expression levels of genes of interest in cork oak or even in other oaks for which large transcriptomics and genomics programs are being developed.

## Supporting Information

Figure S1 **Melting curves generated for all amplicons.**
(TIF)

Figure S2 **Stability values of candidate reference genes calculated by different statistical methods using two data sets.** The ranking of reference genes (*Act*, *CACs*, *EF-1α*, *GAPDH*, *His3*, *Ubq*, *PP2A*, *PsaH*, *Sand*, *β-Tub*) was calculated by geNorm, NormFinder and CV method using the developmental stage and seasonal growth data sets.
(TIF)

Table S1 Amplicon sequences of the 8 candidate reference genes (RG).
(DOC)

Table S2 Amplification efficiencies of the 10 candidate RG measured using the standard curve method.
(DOC)
