# Correction: Reference Gene Selection for Quantitative Real-time PCR Normalization in *Quercus suber*

**DOI:** 10.1371/annotation/13c5a136-9db4-43a9-aad3-f73acb064d0a

**Published:** 2012-09-05

**Authors:** Liliana Marum, Andreia Miguel, Cândido P. Ricardo, Célia Miguel

The first two authors, Liliana Marum, and Andreia Miguel, should be noted as contributing equally to this work.

There was an error in the author contributions. The correct contributions should be: Conceived and designed the experiments: LM AM CPR CM. Performed the experiments: LM AM. Analyzed the data: LM AM CM. Contributed reagents/materials/analysis tools: LM AM CM . Wrote the paper: LM AM CM.

There are errors in references 25,26,27, and 28. The correct references should be:

25. Marum L, Miguel A, Ricardo PC, Miguel C. Identification of GPAT acyltransferases in cork oak; 2011; Arraial d'Ajuda, Bahia, Brazil. BMC Proceedings. pp. P69.

26. Miguel A, Ricardo PC, Jones B, Miguel C. Identification of a putative molecular regulator of cork cambium; 2011; Arraial d'Ajuda, Bahia, Brazil. BMC Proceedings pp. P70.

27. Ramos M, Rocheta M, Carvalho L, Graça J, Morais-Cecilio L. Expression analysis of DNA methyltransferase and co-repressor genes in Quercus suber phellogen: an attempt to correlate with cork quality; 2011; Arraial d'Ajuda, Bahia, Brazil. BMC Proceedings. pp. P169.

28. Paiva JA, Fevereiro P, Marques P, Rodrigues JC, Provost GL, et al. Deciphering cork formation in Quercus suber; 2011; Arraial d'Ajuda, Bahia, Brazil. BMC Proceedings. pp. P172. 

