# Supplementary figures and images for: Reference Gene Selection for Quantitative Real-time PCR Normalization in Quercus suber
Source: PLoS One. 2012 Apr 18;7(4):e35113. doi: 10.1371/journal.pone.0035113 (PMC3329553; doi:10.1371/journal.pone.0035113)

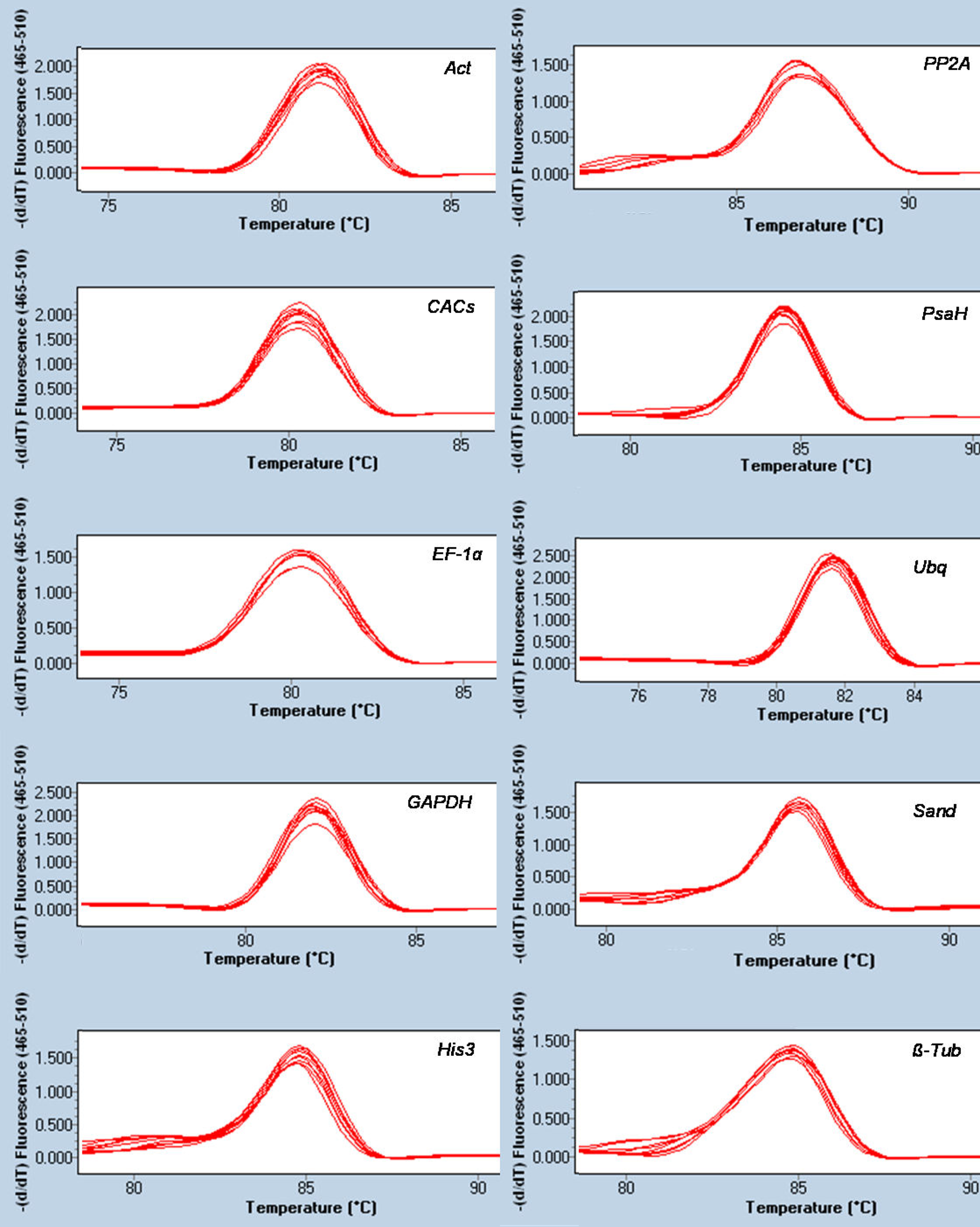

Supplement: Figure S1 — Melting curves generated for all amplicons. (TIF) [file pone.0035113.s001.tif]

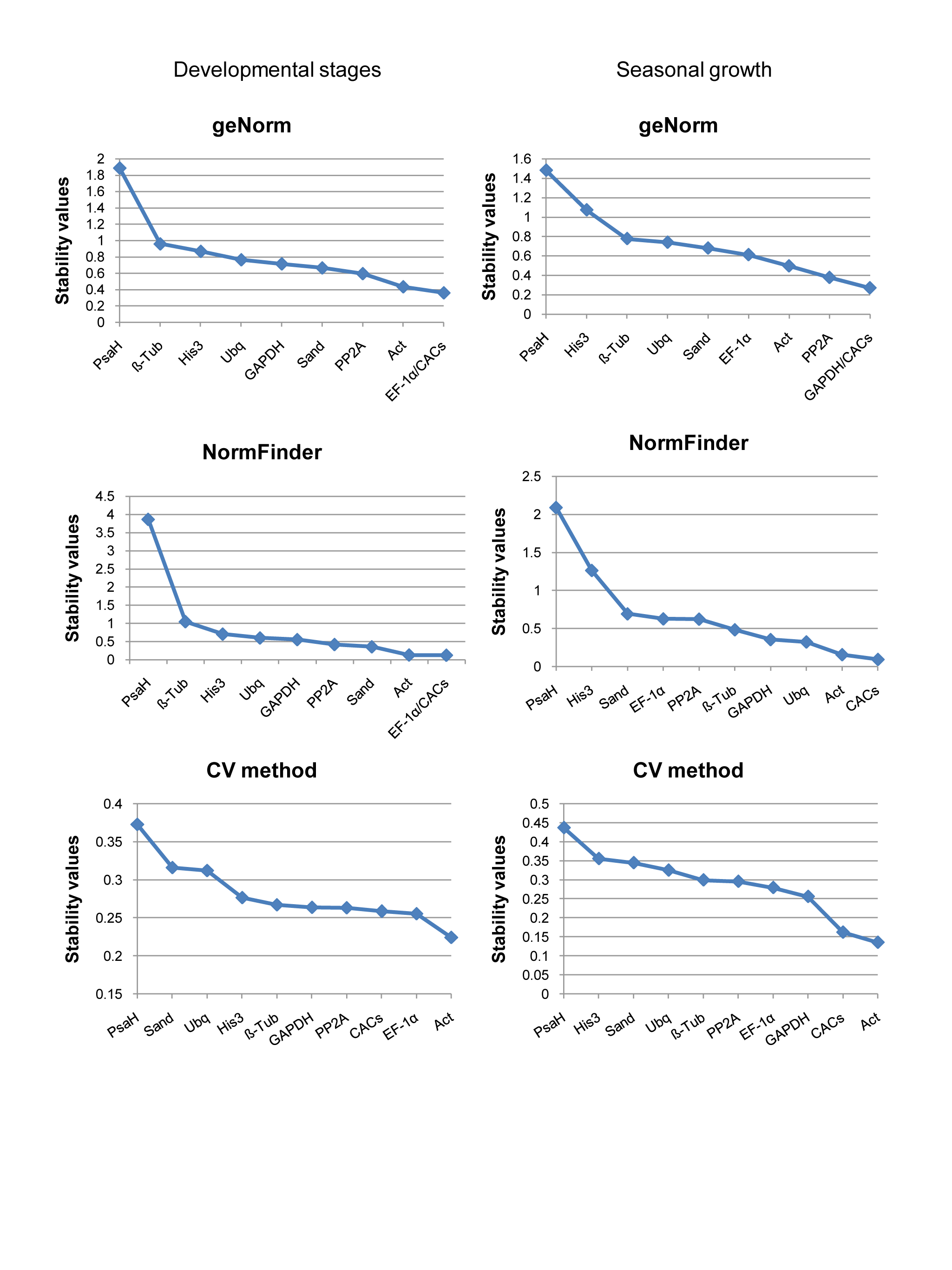

Supplement: Figure S2 — Stability values of candidate reference genes calculated by different statistical methods using two data sets. The ranking of reference genes (Act, CACs, EF-1α, GAPDH, His3, Ubq, PP2A, PsaH, Sand, β-Tub) was calculated by geNorm, NormFinder and CV method using the developmental stage and seasonal growth data sets. (TIF) [file pone.0035113.s002.tif]
