# Supplementary material for: Reference Gene Selection for Quantitative Real-time PCR Normalization in Quercus suber
Source: PLoS One. 2012 Apr 18;7(4):e35113. doi: 10.1371/journal.pone.0035113 (PMC3329553; doi:10.1371/journal.pone.0035113)
Supplement: Table S1 — Amplicon sequences of the 8 candidate reference genes (RG). (DOC) [file pone.0035113.s003.doc]

| **Gene abbreviation** | **Amplicon sequences** |
| --- | --- |
| ***CACs*** | TCTGGGAGAAGAGTGGCTACAATACTGTTGAGTGGGTCCGTTATATTACCAAAGCTGGCTCTTATGAGATTAGGTGCTAGAGACTGGAAATGTTGCTGAGACTATAGATGCCTTAAAATGGGTGGCGATTGAAATATTTGTACGTCTATTTTTCTAAGGATTTGAATGGTGGCTC |
| ***EF-1α*** | TTGTGCCGTCCTCATTATTGACTCCACCACTGGAGGTTTTGAAGCTGGTATTTCTAAGGATGGTCAGACCCGTGA |
| ***GAPDH*** | ACCGACTTCATTGGTGACAGCAGGTCTAGTATATTTGATGCCAAGGCTGGAATTGCATTGAATGACAATTTTGTGAAACTTGTCTCTTGGTATGACAACGAGTGGGGCTACAGTTCCCGTGTGGTTGACCTGATTGTCCACATCGCATCT |
| ***His3*** | GCTCTTCGAGGACACCAATCTGTGCGCCATTCACGCCAAGAGAGTCACCATCATGCCCAAGGATATCCAGCTCGCTCGGAGGATCAGAGGCGAGAGGGCTTA |
| ***PsaH*** | CAGTTGCTCTGAAACCAAGGCCATGGCTTCTCTAGCAACCTTAGCTGCTGTTCAACCAGTCAACATCAAGGGCCTTGGTGGAAGCTCCCTAACAGGAACAAAGCTTGCTATCAAGCCCACTCGCCAGAGCCTAAGGTCCAAAAACTTCAGGACTGGTGCTGTG |
| ***PP2A*** | GAGCCACTCTATCCGATTGCTGTCTTAATTGATGAGCTTAAAAATGAAGATATTCAGCTCCGGCTGAACTCGATCCGCCGGCTCTCTACGATTGCGCGTGCGCTTGGAGAGGAGAGGACCAGGAAGGAGCTGATTCCTTTTCTCAGCGAGAACAATGACGAC |
| ***Sand*** | AGGATTGCAGGATTCGTATTGAAGTGGTCCTTTTGAAGTCAAATGTTCTTAGCGAAGTTCAGAGATCCATGCTAGATGGAGGGATGCATGTTGAAGATTTGCCTACCGATCCATTACCTCGTTCTGGAACTTTATCTCCACATCTGGGSSAACCCAGAGATTCTCTTGAGAGTCTCAAAGAACCATTTGTTGGCATTGGTGGTC |
| ***Ubq*** | CGAAGATCCAGGACAAGGAGGGGATCCCACCGGACCAGCAGAGGTTGATCTTTGCAGGAAAGCAGCTGGAGGATGGCCGCACTCTTGCTGACTACAACATCCAGAAGGAGTCCACCCTTCACCTTGTCCTCCGTCTCCGCGGTGGTGCTTTCTGAGGAATGAAAAAGCCCTG |
