# Supplementary material for: Reference Gene Selection for Quantitative Real-time PCR Normalization in Quercus suber
Source: PLoS One. 2012 Apr 18;7(4):e35113. doi: 10.1371/journal.pone.0035113 (PMC3329553; doi:10.1371/journal.pone.0035113)
Supplement: Table S2 — Amplification efficiencies of the 10 candidate RG measured using the standard curve method. (DOC) [file pone.0035113.s004.doc]

| **RG/**  **Tissue** | ***ACT*** | ***CACs*** | ***EF-1α*** | ***GAPDH*** | ***His3*** | ***PP2A*** | ***Psah*** | ***Ubq*** | ***Sand*** | ***ß-Tub*** |
| --- | --- | --- | --- | --- | --- | --- | --- | --- | --- | --- |
| Leaves | 1.85 | 2.02 | 1.95 | 1.90 | 1.99 | 1.89 | 1.95 | 1.92 | 1.96 | 1.94 |
